# Synthesis and Bioactive Properties of the Novel Coloured Compound Obtained via the Laccase-Mediated Transformation of 5-Aminosalicylic Acid

**DOI:** 10.3390/molecules29061310

**Published:** 2024-03-15

**Authors:** Jolanta Polak, Marcin Grąz, Katarzyna Szałapata, Justyna Kapral-Piotrowska, Kamila Wlizło, Marcin Polak, Anna Jarosz-Wilkołazka

**Affiliations:** 1 Department of Biochemistry and Biotechnology, Institute of Biological Sciences, Maria Curie-Skłodowska University, 20-031 Lublin, Poland; marcin.graz@mail.umcs.pl (M.G.); katarzyna.szalapata@mail.umcs.pl (K.S.); 2 Department of Functional Anatomy and Cytobiology, Institute of Biological Sciences, Maria Curie-Skłodowska University, 20-031 Lublin, Poland; justyna.kapral-piotrowska@mail.umcs.pl; 3 Department of Industrial and Environmental Microbiology, Institute of Biological Sciences, Maria Curie-Skłodowska University, 20-031 Lublin, Poland; kamila.wlizlo@mail.umcs.pl; 4 Department of Zoology and Nature Protection, Institute of Biological Sciences, Maria Curie-Skłodowska University, 20-031 Lublin, Poland; marcin.polak@mail.umcs.pl

**Keywords:** bioactive dye, antimicrobial compounds, biocatalysis, fungal laccase

## Abstract

Biocatalysis processes based on oxidoreductases, such as fungal laccase, are important for discovering new organic compounds with broad structures and potential applications. They include bioactive compounds, which can be obtained through laccase-mediated oxidation of organic substrates having hydroxyl and/or amino groups especially, e.g., 5-aminosalicylic acid (5-ASA) is characterised for its potential for oxidation by a fungal laccase obtained from a *Cerrena unicolor* strain. The biotransformation process was optimised in terms of the buffer and co-solvent concentration, buffer pH value, and laccase activity. Selected crude dyes were analysed for their bioactive properties, toxicity, and suitability for the dyeing of wool fibres. The data obtained clearly indicated that a low concentration of the reaction buffer in the pH range from 5 to 6 and in the presence of 10% acetonitrile increased the rate of substrate oxidation and the amount of the product formed. The red-brown compound obtained via laccase-mediated oxidation of 5-aminosalicylic acid showed antioxidant properties and unique antimicrobial activity against *Staphylococcus aureus* and *Staphylococcus epidermidis* strains with the MIC value of 0.125 mg/mL detected for the purest dye. In addition, it was reported to have good wool fibre dyeing properties and no irritant effect after patch tests on a selected group with increased skin sensitivity.

## 1. Introduction

Most bioactive compounds are natural substances extracted from plants or products from the secondary metabolism of selected microorganisms [1,2]. Many antimicrobial compounds are inspired by natural products and can be obtained in a process of harmful organic synthesis. Nowadays, biocatalysis can play a special role in obtaining new bioactive compounds with novel physicochemical properties that have not yet been described in the literature. Laccase, i.e., an oxidoreductase with low substrate specificity, is the main enzyme studied in terms of the synthesis of novel chemicals via oxidation of aromatic compounds with different structures [3]. Fungal laccase oxidises phenols and organic amine compounds to reactive radicals, which can undergo oligomerisation or non-enzymatic spontaneous coupling reactions into new hybrid molecules, sometimes very difficult to predict, even with a heterocyclic core [4]. The newly formed end-products include compounds exhibiting new features, such as antimicrobial, dyeing, and antioxidant properties [5,6,7,8]. Nevertheless, the discovery of new bioactive compounds with a wide range of activities and applications is desirable in the context of increasing the resistance of microorganisms to commonly used therapeutics.

In the present work, 5-aminosalicylic acid (5-ASA) was examined as a potential substrate for fungal laccase due to the presence of hydroxy and amine groups, the main substituents involved in the oxidation by fungal laccase [9]. 5-ASA was transformed by fungal laccase from the tested *Cerrena unicolor* strain into a novel red-brown coloured compound and analysed to assess its bioactivity and possible application for the dyeing of fabrics. The biotransformation process was optimised especially in terms of the pH value, buffer and co-solvent concentration, and laccase activity. The crude products obtained in different conditions showed antioxidant properties and antimicrobial activity against *Staphylococcus* strains, which have not yet been reported in the literature. Moreover, good wool fibre dyeing properties and no irritant effect after patch testing on a selected group with increased dermal sensitivity were also reported, which confirms the usability of this product as a bioactive dye.

## 2. Results and Discussion

The tested substrate, namely 5-aminosalicylic acid (5-ASA), known as mesalamine or mesalazine, is a registered drug used in therapy for inflammatory bowel disease and ulcerative colitis [10,11]. It possesses both hydroxy and amine groups, which can be oxidised directly by fungal laccase; therefore, it was recognised as a potential substrate for this universal enzyme. It is characterised by a low oxidation potential, which indicates the possibility of its oxidation by fungal laccase (Table 1). The kinetic studies confirmed these findings, and 5-ASA was oxidised by laccase obtained from the culture of *Cerrena unicolor* (LAC) to a red-brown coloured compound with a characteristic wavelength of 500 nm in maximum absorbance, contrary to the absence of the characteristic colour and the confirming peak in the spectrum of the non-oxidised substrate. Moreover, a low value of the *K*_M_ parameter with the concomitant high oxygen demand was noted for this substrate during its oxidation (Table 1).

### 2.1. Optimisation of 5-Aminosalicylic Acid Oxidation

The biotransformation process of organic substrates using LAC into new chemical compounds should be optimised in terms of their efficiency, application safety, and cost-effectiveness. The use of high concentrations of reaction buffers balances the pH value, which is a very important factor during enzymatic biotransformation. In addition, a high concentration of the buffer applied for the biotransformation decreases the content of the compound obtained in the crude non-purified product. Moreover, the addition of a small amount of an organic solvent as a co-solvent can improve the solubility of the substrate and/or the product and can, therefore, enhance the rate of LAC-mediated biotransformation and improve the monitoring of substrate depletion and product formation during the biocatalysis. Also, the optimisation process should provide appropriate conditions for the biocatalysis reaction to be as efficient as possible and the obtained product to be usable without a cost-intensive and lengthy purification process. Therefore, the process of biotransformation was optimised especially in terms of the pH value and the concentration of the buffer and co-solvent present in the reaction mixture.

#### 2.1.1. Influence of pH and Co-Solvent Addition on the Oxidation of 5-ASA

**Figure 1:**
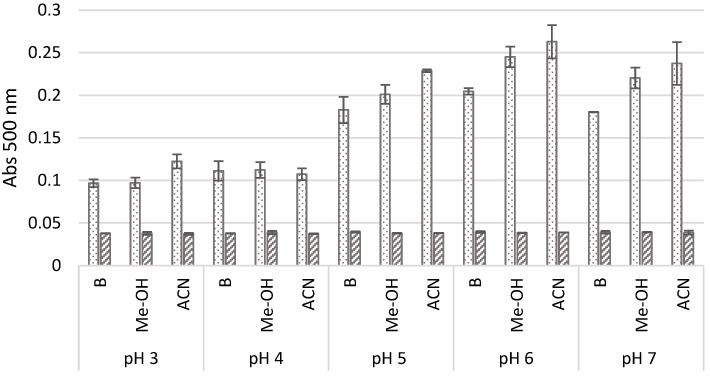
Absorbance of the product obtained after 24 h LAC-mediated 5-ASA oxidation at different pH without (B) and in the presence of selected co-solvents such as methanol (Me-OH) and acetonitrile (ACN) in a final concentration of 10% (*v/v*) (dotted bars, dilution 10×) in comparison to the absorbance of the transformation mixture without laccase addition (striped bars). The differences between groups were statistically significant (ANOVA): B–F_4,10_ = 84.0; *p* < 0.001; MeOH–F_4,10_ = 122.3; *p* < 0.001; ACN–F_4,10_ = 120.2; and *p* < 0.001.

During the first step of the optimisation process, the pH value of the reaction buffer was analysed to assess its influence on the amount of the product expressed as absorbance at 500 nm. When 5-ASA was oxidised in a concentrated buffered mixture and without the addition of the co-solvent, the appearance of a large number of coloured pods was observed after oxidation at pH 3 and 4, and very low amounts were detected when the substrate was oxidised at pH 5 and higher. It was therefore decided to test whether the addition of small amounts of the co-solvent (10%, *v/v*) would prevent product aggregation and, thus, increase product absorbance. The data obtained in all the transformations revealed a bell-shaped profile of the tested substrate, which is characteristic for phenolic substrates oxidised by laccase, with an optimum pH between 5 and 6, regardless of the presence or absence of the co-solvent (Figure 1) [12]. Furthermore, it could be observed that the oxidation in the presence of the selected solvents, especially 10% acetonitrile (ACN), resulted in a higher absorbance of the product, compared to its absorbance obtained without their presence after 24 h of oxidation, which may probably have been caused by the better solubilisation of the products, which are invisible to the naked eye (Figure 1).

#### 2.1.2. Influence of Acetonitrile and Buffer Concentrations on the Oxidation of 5-ASA

**Figure 2:**
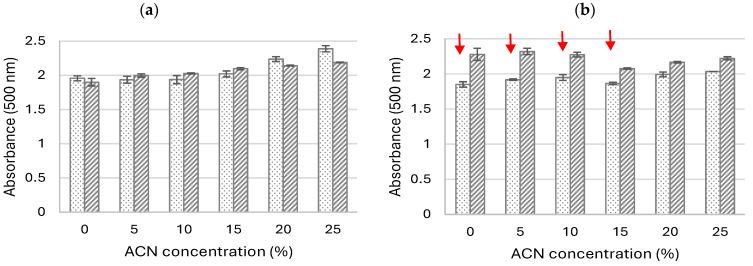
Influence of the acetonitrile (ACN) concentration on the absorbance of 5-ASA oxidation product. After (**a**) 3 h, the differences between groups were statistically significant (test ANOVA): pH 5—F_5,12_ = 51.2; *p* < 0.001; pH 6—F_5,12_ = 50.3; *p* < 0.001. After (**b**) 24 h, the differences between groups were statistically significant (test ANOVA): pH 5—F_5,6_ = 12.9; *p* < 0.005; pH 6—F_5,6_ = 8.4; *p* < 0.05. Absorbance was measured at different pH levels of McIlvaine buffer: pH 5—dotted bars and pH 6—striped bars. Red arrows indicate the presence of the precipitated product.

During the next step of our study, the influence of the acetonitrile and buffer concentrations were examined to select the optimal transformation conditions providing (1) the highest absorbance and (2) best solubility of the product obtained via the LAC-mediated 5-ASA oxidation. The substrate (5-ASA) is not dissolved in acetonitrile and methanol and, as a phenol derivative, can be dissolved in the presence of sodium hydroxide. Hence, its addition to a buffer solution with a well-defined pH value increases the value of the biotransformation mixture. When higher amounts of 5-ASA were introduced to the reaction medium, greater changes in the pH values were noted. Therefore, the optimisation study was carried out using two different pH values of the buffer, i.e., pH 5 and 6 in the presence of different concentrations of acetonitrile. The absorbance of the product was detected after 3 h and 24 h, and the presence of precipitates was monitored at specified intervals. As shown in Figure 2, the presence of ACN had a positive effect on the higher absorbance of the product obtained after 3 h of 5-ASA oxidation, especially in the buffered solution with pH 5. The absorbance of the product noted after 3 h in the mixture without the addition of ACN was more or less the same as the absorbance of products from samples obtained in the presence of 5% and 10% ACN (Figure 2a). Moreover, no visible turbidity of the mixture related to the precipitation of products was observed after 3 h of oxidation, regardless of the pH of the buffer used (Figure 2a). After 24 h of the reaction, visible coloured precipitates were observed in samples synthesised in the presence of the pH 5 buffer and in the presence of ACN in the concentration range from 5% to 15% (Figure 2b). Along with the higher amount of ACN, a lower absorbance of the product was noted when the oxidation occurred at pH 6 (Figure 2b), which may probably have been caused by enzyme denaturation [13]. When the oxidation was run in the presence of a buffer with pH 5, a slightly higher absorbance of the product was obtained at the ACN concentration of approximately 20 and 25% due to the lack of its precipitates. The highest absorbance of the product was obtained at pH 5 after 3 h of the biocatalysis in the presence of 25% ACN, which indicates that the optimal pH for short-term oxidation of 5-ASA is pH 5. In such conditions, the product obtained was not stable and its absorbance was reduced after a longer LAC-mediated oxidation process (Figure 2b).

**Figure 3:**
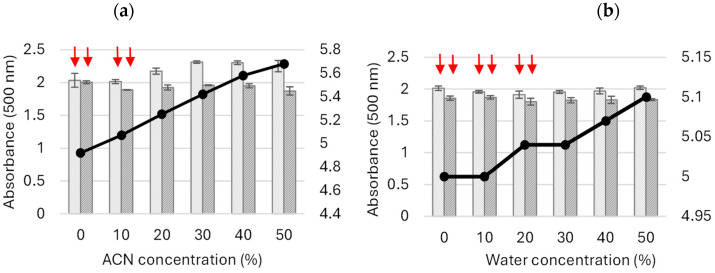
Influence of the buffer dilution obtained through addition of acetonitrile (**a**) (ACN; the differences between groups were statistically significant with ANOVA: ACN 3 h—F_5,18_ = 18.4; *p* < 0.001; ACN 24 h—F_5,18_ = 6.4; *p* < 0.005) and water (**b**) (the differences between groups were statistically significant for water at 3 h—F_5,16_ = 4.2; *p* < 0.05, but not significant for water 24 h—F_5,18_ = 1.4; *p* = 0.29) on the absorbance of the product formed after 3 (dotted bars) and 24 (striped bars) hours of 5-ASA LAC-mediated oxidation in relation to the pH value (black line) of the transformation mixture; red arrows indicate the presence of the precipitated product.

Based on these findings, the next stage of the experiment involved the oxidation of 5-ASA in the presence of different concentrations of ACN and water in the reaction mixture and, consequently, at different concentrations of McIlvaine buffer at pH 5. This pH value allowed maintaining the pH value of the reaction mixture between 5 and 6 through the addition of both ACN and the substrate dissolved at alkaline pH (Figure 3).

The study showed the highest absorbance of the product obtained after 3 h of the transformation process in the presence of ACN as a co-solvent at a concentration higher than 20% (*v/v*). No coloured precipitates were detected in the samples (Figure 3a).

In the case of the mixture containing the water-diluted buffer solution, the absorbance of the formed product was lower, i.e., approximately 1.82 and 1.85 at 500 nm. As in the previous study, a decrease in the product absorbance was observed after 24 h. Moreover, a decreasing buffering capacity of the buffer used was noted as its concentration decreased. Higher pH values of the transformation mixtures were noted in samples containing the higher ACN concentration due to the weakly basic nature of ACN (Figure 3a). In the control experiment using water instead of ACN, the pH values increased slightly together with the decreasing concentrations of the buffer (Figure 3b).

**Figure 4:**
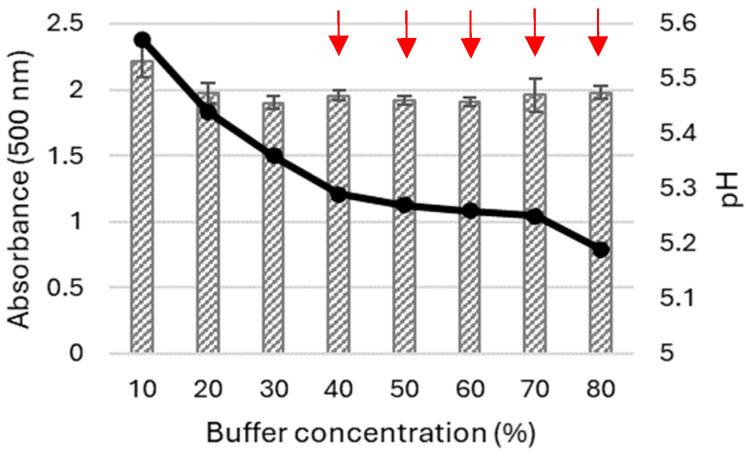
Influence of the McIlvaine buffer concentration on the absorbance of the product obtained after 24 h LAC-mediated oxidation of 5-ASA in the presence of acetonitrile (10%, *v/v*) in relation to the pH value (black line) of the transformation mixture; red arrows indicate the presence of the precipitated product. The differences between groups were statistically significant (ANOVA): F_7,15_ = 5.2; *p* < 0.005.

During the following study, the buffer concentration was optimised for the transformation mixture containing 10% of ACN, and the main selection criteria were the absorbance value of the product obtained and the lack of precipitate (Figure 4). 

As shown in Figure 4, the highest absorbance of the product obtained through the 5-ASA biotransformation was noted in a sample containing both ACN and buffer at a concentration of 10% (Figure 4). The highest pH value was also found in this sample, which may also have increased the solubility of the reaction product.

#### 2.1.3. Influence of Laccase Activity on the Oxidation of 5-ASA

**Figure 5:**
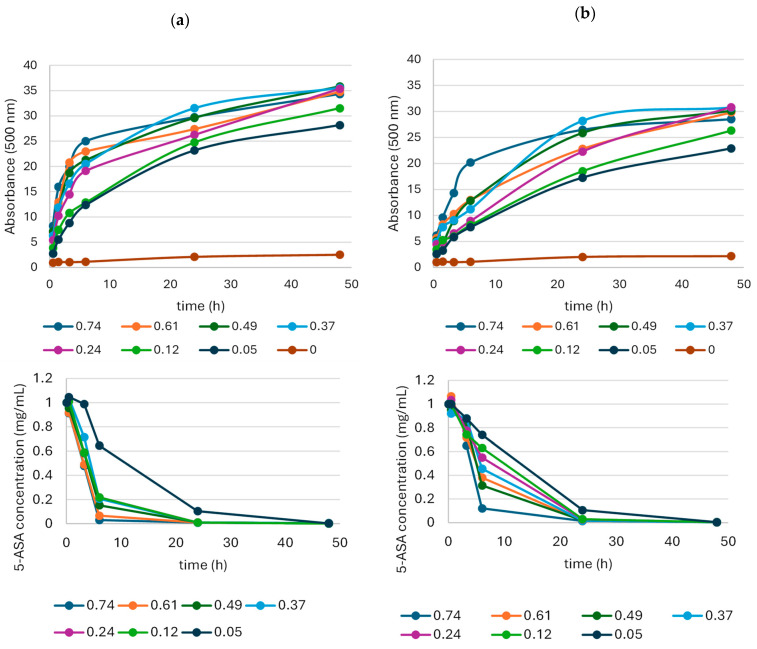
Effect of LAC activity on product absorbance and the substrate depletion rate during the 48 h LAC-mediated 5-ASA oxidation in the presence (**a**) or absence (**b**) of acetonitrile; the legend shows the LAC activity (U per mg of 5-ASA) used in the biotransformation process.

In the next stage of the study, LAC activity was optimised for the oxidation of 5-ASA (final concentration of 1 mg/mL) in a transformation mixture containing a 2% McIlvaine buffer (Figure 5). Such a low concentration of the buffer used ensured both the absence of dye precipitates, a low amount of impurities in the final product, and the stability of a pH level not exceeding the value of 6. Moreover, the oxidation was conducted using 10% ACN as a co-solvent to check its influence on the amount and the homogeneity of the oxidation product analysed during the biocatalysis.

The results showed that the rate of 5-ASA oxidation and the absorbance of the product depended on the LAC activity and the presence of acetonitrile (Figure 5).

The absorbance of the products obtained during the first 6 h of the biotransformation process decreased with the decreasing laccase activity, irrespective of the presence of acetonitrile (Figure 5). The ACN had a positive effect on the 5-ASA oxidation, resulting in the faster depletion of the substrate and a higher absorbance of the products (Figure 5a). At the LAC activity of 0.74 U per mg 5-ASA, the substrate oxidation rate in the presence of ACN was 0.16 mg/h, compared to 0.14 mg/h in the sample without ACN (Figure 5). The substrate oxidation rate at the lowest LAC activity (0.05 U/mg) was 0.038 mg per hour, regardless of the presence or absence of ACN. Among the six tested LAC activities, 0.37 U per mg of 5-ASA seemed to be the optimal value. Despite the slightly lower rate of substrate oxidation, the highest amount of the product expressed as the absorbance at 500 nm was recorded after the 24-h biocatalysis.

**Figure 6:**
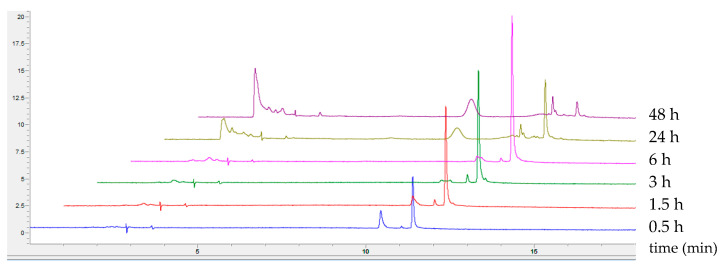
Profile of the elution of 5-ASA biotransformation products obtained in the presence of 10% acetonitrile addition, with changes over time, and detection at 500 nm.

The detailed HPLC analysis of products obtained during the biotransformation process demonstrated the presence of two by-products during the first 30 min of 5-ASA oxidation (t_r_: 10.6 min, 11.3 min). After 24 h, the amounts of these compounds decreased, and new coloured products appeared (t_r_: around 2 min, 8.7 min) (Figure 6).

**Figure 7:**
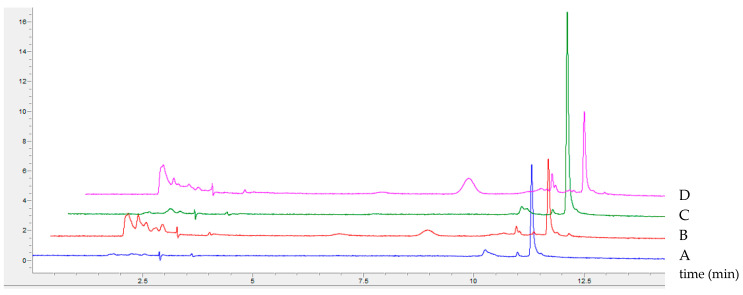
Profile of the elution of biotransformation products obtained after 6 h (lines A and C) and after 24 h (lines B and D) of 5-ASA oxidation mediated by fungal laccase without ACN (lines A and B) and in the presence of 10% ACN (lines C and D).

Similar observations were noted in the case of 2-amine-3-methoxybenzoic acid oxidation, for which four different by-products appeared during the first hours, their quantity decreased after 24 h, and a new stable product was formed via non-enzymatic coupling reaction [14]. Moreover, no difference in the homogeneity of the coloured products was visible, regardless of the presence or absence of ACN (Figure 7).

This shows that acetonitrile does not affect the mechanism of 5-ASA oxidation and the process of spontaneous coupling reactions, but induces a significant increase in the absorbance of the products and the rate of substrate oxidation, which, to our knowledge, has not yet been described in the context of laccase-mediated biotransformation.

### 2.2. Bioactive Properties of 5-ASA Oxidation Products

Compound 5-aminosalicylic acid (mesalamine) is a drug with antimicrobial, chemopreventive, anticancer, anti-inflammatory, and neuroprotective effects leading to [15] its widespread use in medicine. However, this substance also has many features that make its use difficult. 5-ASA is characterized by low solubility in water; it is an easily oxidized and relatively unstable substance, strongly influenced by light, temperature, and pH [15]. The above-described features result in the design and synthesis of new 5-ASA derivatives, which are characterized by increased solubility and absorption, lower cytotoxicity, and enhanced antimicrobial activity [16,17,18,19]. Moreover, one of the proposed paths of application of 5-ASA derivatives is the synthesis of azo dyes that exhibit biological activity [20]. However, none of the publications mentioned relate to the biotransformation of 5-ASA into biologically active compounds using fungal laccase as a catalyst, which was reported in the present study.

Different dyes and a control sample (S and C) were prepared to check their properties, i.e., their most important antimicrobial and antioxidant features. They were obtained in mixtures containing different concentrations of McIlvaine buffer and 5-ASA substrate to check how the concentration of the components of the biotransformation mixture affects the properties of the resulting products. The pH of the transformation mixture ranged from 5 to 6.3. The resulting products were lyophilised and analysed for their selected bioactive properties. The conditions of the synthesis and the acronyms of the tested products are summarised in Table 2.

#### 2.2.1. Antimicrobial Properties

**Figure 8:**
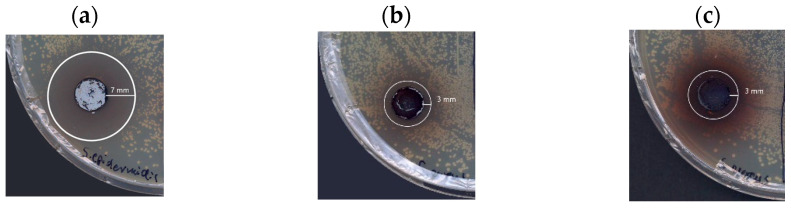
Growth inhibition zones noted for various products of the 5-ASA LAC-mediated transformation: (**a**) *Staphylococcus epidermidis* (Dye16B); (**b**) *Staphylococcus aureus* (Dye16B); and (**c**) *Staphylococcus aureus* (Dye2B).

Literature data provide many examples of antimicrobial agents obtained during the laccase-mediated biotransformation of organic substrates [5]. These mainly include compounds obtained by modifying existing antibiotics, and there are a few examples of compounds obtained de novo [5]. Based on the preliminary diffusion agar test prepared using three reference strains, Gram-negative bacterium *Escherichia coli* ATCC 25922 and Gram-positive bacteria *Staphylococcus aureus* ATCC 25923 and *Staphylococcus epidermidis* ATCC 14990, the transformation products obtained via the oxidation of 5-ASA by fungal laccase were recognised as potential antimicrobial compounds with a clearly marked zone of inhibition for staphylococcal growth (Figure 8). In the case of the transformation product synthesised in the different pH conditions (pH 5 and 6.3), it was observed that the dye synthesised at the lower pH value (Dye16B) showed antibacterial activity against the *S. aureus* strain (3 mm inhibition zone) and *S. epidermidis* (7 mm inhibition zone) in contrast to the dye synthesised in the environment with the higher pH value (Dye30B), which did not show any antibacterial activity (Table 3).

The lack of antimicrobial activity in the sample synthesised at the higher pH may be due to the higher content of impurities (buffer ingredients and NaOH), which ‘dilute’ the antimicrobial effect of the resulting compound (Table 2). The *S. epidermidis* strain exhibited higher sensitivity to the tested dyes with the inhibition zone approximately twice as much as the inhibition zones noted for the growth of the *S. aureus* strain (Table 3). 

In our study, no antibacterial activity was observed for 5-ASA (S) and the control sample containing lyophilised buffer solutions with LAC (C), which is contrary to the literature data [15]. Its antibacterial effect, i.e., the reduction in the occurrence of unfavourable amounts of *Escherichia-Shigella* bacteria in the intestines of sick patients, has been confirmed as well [21]. Mesalamine has also been confirmed to inhibit the growth of *E. coli* at a concentration of 100–200 ug/mL [22]. In contrast, laccases isolated from fungi *Cerrena unicolor* and *Trametes hirsuta* have growth-inhibiting properties in the case of *E. coli* and *S. aureus* strains [23,24]. Antibacterial activity was also confirmed for *Bacillus subtilis* laccase, which inhibited the growth of the *E. coli* strain [25]. Such findings demonstrated that new coloured products exhibited antimicrobial properties, which are not associated with the properties of LAC and 5-ASA.

The results obtained from the agar diffusion test were confirmed in submerged cultures of bacteria cultivated with the other dye samples obtained in the presence of the different concentrations of buffer. Transformation products (Dye2B, Dye16B, and Dye20B) showed activity against the *S. aureus* strain and the *S. epidermidis* strain (Table 4). 

Greater sensitivity to the dyes mentioned above was observed in the case of the *S. epidermidis* strain, where the MIC values for three of them ranged from 0.125 mg/mL to 0.5 mg/mL (Table 4). Moreover, in the case of these samples, the MBC parameter was also possible to calculate, with a value of 0.5 mg/mL and 1 mg/mL, depending on the sample. In the case of the *S. aureus* strain, more significant differences in the antibacterial activity were found. The highest activity was demonstrated by Dye2B with a MIC value of 0.125 mg/mL and a MBC value of 0.6 mg/mL. Dye16B had lower activity with a MIC value of 0.5 mg/mL and a MBC value of 0.6 mg/mL. The weakest antibacterial activity was observed for Dye20B, for which the MIC value was 0.9 mg/mL. In contrast, the MBC value was not determined in the tested concentration range (MIC > 1 mg/mL). It is worth emphasising that, in the case of the higher concentration of the buffer used for the synthesis, the antimicrobial activity of the transformation products was lower because of contaminations present in the lyophilised dye. Even so, the crude dyes obtained via the 5-ASA oxidation can be effective antimicrobial compounds against staphylococci (Table 4). 

Some publications describe the antimicrobial properties of different 5-ASA derivatives against various bacterial strains. The lack of sensitivity of Gram-negative bacteria (*Pseudmonas aeruginosa*) and the low sensitivity of Gram-positive bacteria (*Bacillus subtilis*) to palladium Schiff base complexes derived from aminosalicylic acid were also observed by Klaus et al.; however, the very poor solubility and stability of the obtained compounds excludes these compounds from the potential application as antimicrobial substances [26]. Similar conclusions were drawn from research conducted by Shehab and Rasheed [27], where it was observed that aldehyde derivatives obtained using the microwave method showed reduced antimicrobial activity against Gram-negative bacteria (*Proteus mirabilis*) and higher antimicrobial activity against Gram-positive bacteria (*Enterococcus faecalis*), at much higher effective concentrations of 5–15 mg/mL. On the other hand, complete inhibition of the growth of *S. aureus* and *E. coli* strains was demonstrated by silver nanoparticles supported by functionalized hydroxyapatite with 5-ASA after the first 4 h of incubation in the concentration range of 0.5–2 mg/mL [28]. Great antimicrobial activity was also demonstrated by derivatives obtained by Jasim and co-workers [19], which caused a 23 mm diameter growth inhibition zone of *E. coli* (5-(((1H-pyrrol-2-yl)methylene)amino)-2-hydroxybenzoic acid) and a 25 mm diameter growth inhibition zone of *S. aureus* (5-(((1H-indol-2-yl)methylene)amino)-2-hydroxybenzoic acid) at a concentration of 1 mg/mL. 

#### 2.2.2. Antioxidative Properties

The antioxidant properties of the various dyes and control compounds were tested in the ABTS radical reduction test. Substrate 5-ASA exhibited strong antioxidant potential, even more significant than that of the dyes, associated with the presence of hydroxy groups in the molecules, which agrees with the typical antioxidative properties of phenols [29]. The unpurified dyes showed antioxidative potential comparable to that in the control sample containing lyophilised ingredients of McIlvaine buffer, such as citric acid and a small amount of LAC (Table 5). Such results do not prejudge the absence of such properties in the purified compound. Stronger antioxidant activity was observed along with the lower content of impurities, which was especially evident in the case of the Dye2B sample. Antioxidant properties were also demonstrated by 5-ASA derivatives obtained in the studies of Perez et al. [30]. These 5-ASA amide derivatives showed an increased ability to scavenge free radicals compared to the control 5-ASA preparation, which was examined using two research methods (DPPH and ABTS assays). It was confirmed that compounds 2-((3-carboxy-4-hydroxyphenyl)carbamoyl)-4-fluorobenzoic acid and 5-[(2-carboxybenzoyl)amino]-2-hydroxybenzoic acid caused the 100% reduction in ABTS radicals at concentrations above 0.102 mM, while other derivatives showed the ability to scavenge approximately 60% of ABTS free radicals at the same concentration.

### 2.3. Toxicity of Substrate and Oxidation Products

The synthesis process and chemicals used should be safe for both the user and the environment. Therefore, cytotoxicity, as well as environmental toxicity, was evaluated in selected samples. The study showed that the substrate and the buffer control sample showed low toxicity to a normal fibroblast cell line (Table 6). Depending on the sample tested, different levels of toxicity of the dyes were observed. As the amount of impurities in the samples decreased, the value of the IC50 parameter also decreased, indicating the increasing toxicity of the dyes (Table 6). On the other hand, research conducted by Yousefi et al. showed the lack of cytotoxicity of the new 5-ASA derivatives (glucose and xylitol esters or fatty acids derivatives) simultaneously increasing the anti-inflammatory properties in comparison to 5-ASA [17,18]. Reduced cytotoxicity was also demonstrated by galactose and fructose ester derivatives, which were characterized by reduced cytotoxicity compared to the original drug [31].

The environmental studies also revealed high toxicity of the product obtained in the mixture containing 20% buffer, which confirms that the product obtained in this way can negatively affect the marine bacterium *Vibrio fisheri* (Table 7). These data confirm our previous observations, i.e., compounds with antimicrobial properties also show increased environmental toxicity [23,32].

### 2.4. Dyeing and Irritating Properties of the 5-ASA Oxidation Product

The product obtained via the LAC-mediated transformation of 5-ASA in the transformation mixture with pH 6 was a red-brown coloured dye with good wool fibre dyeing potential (Table 8). The dyed fabrics tested for colour fastness had high resistance to dry and wet rubbing and resistance to washing at 40 °C (index 4–5). The fibres exhibited lower resistance to light and acid sweat (Table 8).

The dye was tested for allergenic potential to confirm its safety. None of the volunteers in the study had an allergic reaction after skin contact, confirming that the dye can be used on sensitive skin and by subjects with an increased allergic history (Table 9).

## 3. Materials and Methods

### 3.1. Chemicals and Co-Solvents

Chemicals, i.e., tartaric acid and 5-aminosalicylic acid (5-ASA), were purchased from Aldrich (Wuxi, China). Ammonium bicarbonate and 2,2′-azino-bis(3-ethylbenzthiazoline-6-sulfonic acid (ABTS) was supplied by Sigma (Livonia, MI, USA). All chemicals were of analytical grade and were used without further purification. Organic solvents were purchased from POCH (Gliwice, Poland) and were HPLC-grade.

### 3.2. Laccase Assay

The white rot fungus *Cerrena unicolor* was the source of extracellular laccase (LAC). The strain (collection number FCL139) was obtained from the Fungal Collection of the Department of Biochemistry and Biotechnology of Maria Curie-Skłodowska University, Lublin (Lublin, Poland). LAC was obtained and purified using a procedure described previously by Luterek and co-workers [34].

LAC activity was determined using ABTS as a substrate. A volume of 50 µL of the LAC sample was added to the reaction mixture containing 200 µL of ABTS (2.5 mM final concentration) suspended in 750 µL of 0.1 M sodium-tartrate buffer with pH 3. The oxidation of ABTS was monitored spectrophotometrically for one minute at 414 nm (λ_414_ = 36 048 M^−1^ cm^−1^) using a Cary 50 Bio spectrophotometer (Varian, Palo Alto, CA, USA). The LAC activity was expressed in U/mL, where one unit (U) of the enzyme oxidised 1 µmol of ABTS per 1 min at 25 °C. The purified laccase used in both the optimisation and large-scale dye synthesis stages had a starting activity of 24.6 U/mL (specific activity—930 U/mg) and was then added to the transformation mixture in a volume that achieved the desired final activity.

### 3.3. Substrate Characterisation

*Oxygen uptake* was detected during the 5-ASA transformation by LAC with a biological oxygen monitor (YSI model 5300). The standard vessel contained 3 mL of the transformation mixture containing 1 mM of 5-ASA dissolved in 0.1 M sodium-tartrate buffer with pH 4.5. Each measurement was carried out for 3 min of the transformation and the oxygen uptake was calculated in nmol O_2_/mL/min according to Bernhardt [35].

*The cyclic voltammetry* measurement of the 5-ASA substrate was carried out according to the procedure described previously [21] using a µAUTOLAB type III potentiostat/galvanostat (Metrohm Autolab B.V., Utrecht, the Netherlands). The measured potential recorded vs. the Ag/AgCl/KCl_sat_ electrode was corrected by +0.199 V to the normal hydrogen electrode (NHE).

*The kinetic constants K_M_* of 5-ASA were monitored at a wavelength of 500 nm in 0.1 M sodium-tartrate buffer with pH 4.5 using a Cary 50 Bio spectrophotometer (Varian, Palo Alto, CA, USA) and were calculated using the Lineweaver–Burk equation.

### 3.4. Biotransformation of Substrate 5-ASA by Fungal Laccase—Optimisation Process

*The influence of buffer pH and the addition of organic solvents* was determined for a 1 mM final concentration of 5-ASA soluble in a mixture containing 100 mM tartrate buffer with a pH value in the range from 3 to 7, acetonitrile and methanol (10% final concentration, *v/v*), and laccase with the final activity of 1 U/mL. The absorbance of the product was monitored at 500 nm using a multiplate reader for a maximum of 24 h (Spark, Tecan, Grödig, Austria). Tests were conducted in duplicate or triplicate and are presented as mean ± SD.

*The buffer and acetonitrile concentration optimisation steps* were prepared for a 0.1 mg/mL final concentration of 5-ASA and was carried out in a transformation mixture containing McIlvaine buffer with pH 5 and pH 6, different concentrations of ACN or distilled water, and laccase with the final activity of 0.8 U/mL. The absorbance of the product was monitored at 500 nm using a multiplate reader for a maximum of 24 h (Spark, Tecan, Austria). Tests were conducted in 2–4 replicates and are presented as mean ± SD.

Substrate 5-ASA in a 1 mg/mL final concentration was oxidised in a 1 mL transformation mixture by LAC with different final activities ranging from 0.05 to 0.74 per milligram of 5-ASA at room temperature, 22 ± 3 °C. The final concentration of acetonitrile was 10% (*v/v*) and the final concentration of McIlvaine buffer was 2% (*v/v*) of the original concentration of the buffer with pH 3. The starting pH value of the transformation mixture was 5.8. The absorbance and spectrum of the product was monitored at 500 nm using a multiplate reader for a maximum of 48 h (Spark, Tecan, Austria). Tests were conducted in triplicate and are presented as mean ± SD. A single sample of each variant was additionally analysed using HPLC.

*The large-scale synthesis of the dye* was performed in a 250 mL screw-top glass bottle containing 0.1 L of the reaction mixture. The transformation mixture contained the 5-ASA substrate in a final concentration of 0.6 mg/mL or 1 mg/mL dissolved in ultrapure water with the addition of 1 M NaOH. The final concentration of the McIlvaine buffer in the reaction mixture varied from 2% to 30% (*v/v*), and the pH value was in the range from 5.09 to 6.34. The final activity of laccase added to the reaction mixture was 0.8 U/mL. The transformation was carried out in a rotary shaker (120 RPM) at room temperature (22 °C) for 24–48 h. The absorbance of the synthesised dye was measured spectrophotometrically at 500 nm, and the transformation process was ended at the moment of plateau. The dye was further lyophilised and stored at 4 °C until use.

### 3.5. HPLC Analysis of the Transformation Mixture

The biotransformation process was monitored by high performance liquid chromatography using RP-HPLC with a Synergi Hydro-RP column (25 mm × 4.6 mm, 4 µm) coupled with a DAD detector (Agilent 1260 Infinity, Agilent^®^, Santa Clara, CA, USA). Elution was performed in the gradient mode from 10 to 50% acetonitrile (ACN, eluent B) over 10 min. Ammonium bicarbonate (10 mM) with pH 7.5 was used as eluent A. 5 min elution of 10% ACN was used before the gradient step, and 3 min elution of 50% ACN was performed after the gradient step. After each analysis, a 5 min post run was performed with 10% of eluent B to restore the initial conditions of the analysis. The eluent flow rate was maintained at 1 mL/min throughout the separation process and the temperature of the column was maintained at 22 °C. Each 2 µL sample was injected using an autosampler. The elution of the separated compound was carried out at 210 nm and 500 nm. Agilent OpenLAB CDS ChemStation LC and CE Drivers (A.02.10 (026) version) software was used for data processing and reporting. Identification of the substrate peak was achieved by comparing retention times with the standard.

### 3.6. Properties of Dye

#### 3.6.1. Antimicrobial Properties

*AGAR plate diffusion test.* The agar plate diffusion test of the 5-ASA substrate and its transformation products was carried out according to the CLSI Protocol using *E. coli* ATCC^®^ 25922^TM^, *S. aureus* ATCC^®^ 25923^TM^, and *S. epidermidis* ATCC^®^ 14990^TM^ strains [36,37]. The *inoculum* for the diffusion tests was prepared by transferring a small amount of biological material from a fresh agar plate to a Falcon tube containing 10 mL of sterile distilled water to obtain a suspension with an optical density corresponding to the 0.5 McFarland. Then, the appropriate dilution was performed to obtain a suspension with an approximate density of 10^6^ cfu/mL. Petri dishes with Mueller–Hinton agar medium and wells with a 1 cm diameter were used for the diffusion tests. Each plate was inoculated with 50 µL of the bacterial suspension spread evenly over the entire surface of the plate with a sterile bacteriological spreader. Then, 100 µL of the active substance preparation was added to the wells and left at room temperature for about 20–30 min for full absorption into the agar. After this time, parafilm-protected plates were transferred to a laboratory incubator and incubated for 24 h at 37 °C. After incubation, the plates were removed from the incubator and the zones of growth inhibition were read. The result was presented as the radius of the growth inhibition zone calculated from the edge of the well.

*MIC and MBC values determination.* The Minimal Inhibitory Concentration (MIC) and the Minimal Bactericidal Concentration (MBC) of the 5-ASA substrate and its transformation products were determined according to the CLSI Protocol using *S. aureus* ATCC^®^ 25923^TM^ and *S. epidermidis* ATCC^®^ 14990^TM^ strains [38]. The tested compounds were dissolved in Mueller–Hinton Bullion to the final concentration range from 0.031 to 1 mg/mL. In each well of a 96-well plate, 200 µL of the mixture was inoculated with 10 µL of the bacterial culture (final density ~ 10^2^ cfu/mL). After 24 h of incubation at 37 °C, the growth of the bacteria was controlled, and the MIC values were determined on the basis of the absence of bacterial growth by observation with the unaided eye. For determination of the MBC value, 10 µL of the suspension from each well was transferred to a fresh sterile medium and incubated for another 24 h at 37 °C. The lack of turbidity indicated bactericidal activity of the investigated substances, and the MBC value was, thus, determined. All analyses were performed in triplicate.

#### 3.6.2. Antioxidant Properties

The ability of the tested dye to reduce the dark green ABTS radical cation (ABTS^●+^) was evaluated in this assay. ABTS was first dissolved in the final concentration of 7.4 mM in 5 mM phosphate buffer with pH 7.4 and oxidised by reacting with K_2_S_2_O_8_ (2.4 mM final concentration) in a dark bottle at room temperature for 16–18 h. The ABTS^●+^ solution was diluted with PBS to an absorbance of 0.65 at 734 nm measured using a Tecan plate reader (BioTek). ABTS^●+^ radical cation (990 µL) was added to the tested dye sample (10 µL) in a 96-well plate and incubated for 10 min at room temperature. The concentration of the tested compounds was ranged from 10 mg/mL to 0.01 mg/mL. Simultaneously, the negative control (*X_C_*) and the control solution of dyes for colour correction (*X_D_*) were prepared. The change in the absorbance of ABTS^●+^ with the dye sample (*X_P_*) and the controls were measured in the Tecan plate reader at 734 nm, and the ABTS^●+^ reduction rate (*A_R_*) was calculated according to the following equation:

(1) $$A_{R}=\left(\frac{X_{C}-\left(X_{P}-X_{D}\right)}{X_{C}}\right)\times 100\%$$

All analyses allowing determination of antioxidative properties were performed in triplicate at room temperature, 22 ± 3 °C. The antioxidative potential of the tested compounds was expressed as IC_50_, which represents the final concentration of the tested compound (µg/mL) that reduces 50% of the radical amounts.

#### 3.6.3. Toxicity

##### Cytotoxicity

*Cell cultures.* Human dermal fibroblasts (HDFa) were acquired from the American Type Culture Collection (ATCC, Manassas, VA, USA) and cultured in a humidified atmosphere of 5% CO_2_ at 37 °C in Dulbecco’s modified Eagle’s medium (DMEM) supplemented with 10% foetal bovine serum (FBS) and antibiotics (100 IU/mL penicillin and 100 µg/mL streptomycin). Cells at the density of 1 × 10 ^5^ cells/mL were seeded on 96-well plates. After a 24 h incubation, the medium was replaced with a fresh one with 2% of FBS supplemented with the tested samples or not (control).

*MTT assay*. The number of viable cells was estimated by the ability of mitochondria to convert yellow dye 3-(4,5-dimethylthiazo-2-yl)-2,5-diphenyl-tetrazolium bromide (MTT) to purple formazan crystals. Human dermal fibroblasts were cultured on 96-well plates and treated with the tested samples for 24 h. After this time, 25 µL of MTT (5 mg/mL) was added to each well and incubated for 4 h in a 5% CO_2_ humidified atmosphere at 37 °C. Then, 100 µL of a 10% SDS solution in 0.01 N HCL was added to dissolve the formazan crystals. The absorbance was measured at 570 nm using a microplate reader (BioTek 800 TS, Agilent Technologies, Santa Clara, CA, USA).

*Lactate dehydrogenase (LDH) assay*. The LDH kit is based on the measurement of lactate dehydrogenase (LDH) released to the medium by damaged cells. The amount of LDH released to the medium was assessed using the LDH-Cytotoxicity Assay Kit (BioVision, USA) according to the manufacturer’s instructions. Cells were grown on a 96-well plate and incubated with the tested samples for 24 h. Next, 10 µL of clear medium solutions from each well were transferred to a new 96-well multiplate. 100 µL of the LDH Reaction Mix was added to each well and incubated for 30 min in darkness at room temperature. The reaction was stopped by adding 10 µL of stop solutions. Absorbance was measured with a microplate reader (BioTek 800 TS, Agilent Technologies, Santa Clara, CA, USA) at 490 nm with a reference wavelength at 690 nm. Results obtained from three independent experiments were calculated according to the formula

Cytotoxicity (%) = Test Sample − Low Control)/(High Control − Low Control) × 100%(2)

All analyses were performed in triplicate and presented as mean ± SD. The IC50 value was calculated by nonlinear regression using a curve fitting program in GraphPad Prism 5.0.

##### Environmental Toxicity

The environmental toxicity of the 5-ASA substrate and the dye was assessed using the 81.9% Basic Test Microtox Protocol (https://www.modernwater.com/assets/Technical%20Support/Toxicity/Manuals/ACUTE%20User%27s%20Manual.pdf, accessed on 1 September 2018) and apparatus Microtox Model 500. The toxicity was expressed as the half maximal effective concentration (EC50) according to the 81.9% Basic Test Microtox protocol. All reagents and the *Vibrio fisheri* bacterium were purchased from Tigret (Polska), the Polish representative of Modern Water company (New Castle, DE, USA). All analyses were performed in triplicate and presented as mean ± SD.

#### 3.6.4. Dyeing Properties

Wool fabric dyed using a 1% solution of the crude dye was tested to assess its colour stability during the fastness test prepared and analysed by the TKANLAB Laboratory (Łódź, Poland). The fibre characteristics were as follows: yarn linear mass—warp R63 ± 4/2, weft R74 ± 4/2; number of threads per 10 cm—warp 175 ± 10, weft 135 ± 8; mass per unit area—215 ± 10 g/m^2^; and fat content—0.8 ± 0.3%.

#### 3.6.5. Allergenicity Patch Test

The study was conducted in accordance with Regulation No 1223/2009 of the European Parliament and of the Council (EC) of 30 November 2009 on cosmetics and with the recommendation of Cosmetics Europe—The Personal Care Association Guidelines [39,40]. The research was carried out in compliance with the internal procedures of Dr. Koziej Institute Ltd. (Warsaw, Poland). described previously [32]. The allergenic and irritant features were assessed to determine local skin tolerance to the crude non-purified dye in a group of 50 skin-healthy volunteers aged 19–70 with increased sensitivity and positive allergenic screening of the skin. Two kinds of samples were prepared: the first was the dye diluted to 1% and applied to the patch and the second was material stained with the tested dye cut into the size of the chamber and placed on the patch. The study was conducted using *Finn Chambers on Scanpor* 100 *×* 10 8 mm. The whole patch was then applied to the volunteer’s skin (shoulders, arm, or back). The patches were removed after 15 min. The patch tests were assessed after 48 and 72 h after patch removal.

#### 3.6.6. Statistical Analysis

The means are given together with their standard deviations, ± SD. In the statistical analyses, parametric test was applied after checking whether the distribution was consistent with the normal distribution (Kolmogorov–Smirnov test; all cases *p* > 0.05). A bilateral critical region was assumed in the tests, and results were deemed significant if the probability of committing an error of the first kind was equal to or less than 0.05. The differences between groups were analysed using an analysis of variance (ANOVA). The calculations were made in STATISTICA 13.3 packet for Windows 10 software [41].

## 4. Conclusions

Fungal laccase is a versatile biocatalyst used for the conversion of various organic compounds into new colour products with application potential. The reaction products include compounds with antioxidant and antimicrobial potential such as the non-toxic and non-allergenic textile dyes described in this paper. Biocatalysis using fungal laccase is an environmentally friendly process and does not require extreme pH and temperature. The presence of a small amount of acetonitrile as a co-solvent increased the solubility of the reaction products, which facilitated the monitoring of the biocatalysis process without affecting the properties of the resulting product. In addition, the use of low concentrations of the reaction buffer minimised the precipitation of the product and reduced the level of impurities and, consequently, increased the antimicrobial potential of the reaction product. The bio-dye obtained using laccase as a reaction biocatalyst does not require a tedious and costly purification process and is ready for use as a bioactive textile dye or a biocidal treatment of materials in the textile industry.

## Figures and Tables

**Table 1:** Characteristics of the selected catalytic parameters of 5-aminosalicylic acid (5-ASA) in terms of its LAC-mediated oxidation.

Structure	*Eo* vs. NHE (V)	Spectrum of Product	λ max (nm)	*K*_M_ (mM)	Oxygen Demand (nM/min/mL)
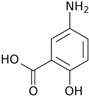	0.494 0.734	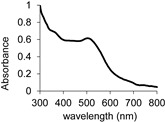	500	0.49	1017

*Eo* vs. NHE—the measured potential was recorded vs. the Ag/AgCl/KCl_sat_ electrode and corrected by +0.199 V to the normal hydrogen electrode (NHE).

**Table 2:** Characteristics of products obtained in different conditions and tested in terms of their bioactive properties and toxicity.

Acronym of Tested Compound	Final Concentration of Buffer before Lyophilisation (%)	Concentration of Salt (M) before Lyophilisation	5-ASA (mg/mL)	pH of Mixture	Content of Impurities in Dry Product (%)
S	0	0	33	9.5	0
C	20	0.04	0	5	100
Dye30B	30	0.06	1	6.35	88
Dye20B	20	0.04	0.6	5.33	89
Dye16B	16	0.032	1	5.09	48
Dye2B	2	0.004	0.6	5.26	51

“S”, substrate 5-ASA; “C”, control sample (lyophilised buffer and LAC).

**Table 3:** Antimicrobial activity against *Escherichia coli*, *Staphylococcus aureus*, and *Staphylococcus epidermidis* reference strains determined in the agar plate diffusion test. The results are presented as the average radius of the growth inhibition zone calculated from the edge of the well [mm].

Tested Compound	Inhibition Zone [mm]		
	*E. coli* ATCC^®^ 25922^TM^ 0.2 × 10^6^ cfu/mL	*S. aureus* ATCC^®^ 25923^TM^ 1.4 × 10^6^ cfu/mL	*S. epidermidis* ATCC^®^ 14990^TM^ 0.8 × 10^6^ cfu/mL
S	-	-	-
C	-	-	-
Dye30B	-	-	-
Dye16B	-	3	7
Dye2B	-	3	6.5

“-“, no inhibition of bacterial growth in the tested concentration; “S”, substrate 5-ASA; “C”, control sample (lyophilised buffer and LAC).

**Table 4:** Summary of the MIC and MBC values of the 5-ASA substrate and its coloured transformation products against staphylococcal strains *Staphylococcus aureus* and *Staphylococcus epidermidis*.

Tested Sample	*Staphylococcus aureus* 2.96 × 10^2^ cfu/mL		*Staphylococcus epidermidis* 5.19 × 10^2^ cfu/mL	
	MIC (mg/mL)	MBC (mg/mL)	MIC (mg/mL)	MBC (mg/mL)
S	-	-	-	-
C	-	-	-	-
Dye30B	-	-	-	-
Dye20B	0.9	>1	0.5	1
Dye16B	0.5	0.6	0.125	0.5
Dye2B	0.125	0.6	0.125	0.5

“-“, no inhibition of bacterial growth in the tested concentration range; “S”, substrate 5-ASA; “C”, control sample (lyophilised buffer and LAC).

**Table 5:** Antioxidative effect of tested samples.

Tested Sample	IC_50_ (µg/mL)
S	1.3
C	19.2
Dye30B	43
Dye20B	31.5
Dye16B	15.4
Dye2B	8.0

“S”, substrate 5-ASA; “C”, control sample (lyophilised buffer and LAC).

**Table 6:** Cytotoxicity of transformation compounds according to MTT and LDH tests.

Tested Sample	IC_50_ (µg/mL)	
	MTT	LDH
S	1285	1125
C	1041	1321
Dye30B	550	721
Dye20B	781	796
Dye16B	906	833
Dye2B	146	196

“S”, substrate 5-ASA; “C”, control sample (lyophilised buffer and LAC).

**Table 7:** Environmental toxicity of the product after 5 and 15 min of exposure according to the Microtox protocol expressed as IC_50_ (µg/mL).

Tested Sample	5 min	15 min
S	55	63
Dye20B	112	95

“S”, substrate 5-ASA.

**Table 8:** Wool fibre dyeing properties of the product and colour fastness of dyed fibres exposed to various factors according to ISO standards [33].

Photo of Dyed Wool	Tested Parameters of Colour Fastness		Scale *	ISO Standard [33]
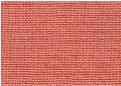	Artificial light ^(1)^	(a)	3	PN-EN ISO 105-B02:2014-11
	Distilled water ^(2)^	(a)	3–4	PN-EN ISO 105-E01:2013-06
		(b)	2	
		(c)	3	
	Washing 40 °C ^(2)^	(a)	4	PN-EN ISO 105-C06:2010
		(b)	4–5	
		(c)	4–5	
	Alkaline sweat ^(2)^	(a)	3–4	PN-EN ISO 105-E04:2013-06
		(b)	2	
		(c)	3–4	
	Acidic sweat ^(2)^	(a)	3–4	PN-EN ISO 105-E01:2013-06
		(b)	1–2	
		(c)	2–3	
	Dry rubbing ^(2)^	(b)	4–5	PN-EN ISO 105-X12:2005
	Wet rubbing ^(2)^	(b)	4	PN-EN ISO 105-X12:2005

* Colour fastness according to a blue (1) or grey (2) scale in which index “8” or “5”, respectively, means the highest resistance and “1” means the lowest resistance according to PN-EN20105-A02:1996 and PN-EN 20105-A03:1996 standards; (a) is the change in the colour of the tested sample; (b) is the soiled whiteness of the accompanying fabric—cotton; and (c) is the soiled whiteness of the accompanying fabric—wool.

**Table 9:** Results of the allergenicity test of the dye obtained through the transformation of 5-ASA.

Characteristics of the Volunteer				Results of the Test		Characteristics of the Volunteer				Results of the Test	
N^o^	Gender	Age	Skin Type	48 h	72 h	N^o^	Gender	Age	Skin Type	48 h	72 h
**1**	M	33	S	**-**	**-**	**26**	M	65	S	**-**	**-**
**2**	F	28	S	**-**	**-**	**27**	F	70	S	**-**	**-**
**3**	F	50	S	**-**	**-**	**28**	M	40	S	**-**	**-**
**4**	F	54	S,A	**-**	**-**	**29**	F	37	S	**-**	**-**
**5**	F	42	S	**-**	**-**	**30**	M	40	S	**-**	**-**
**6**	F	39	S	**-**	**-**	**31**	F	20	S	**-**	**-**
**7**	F	26	S	**-**	**-**	**32**	M	67	S	**-**	**-**
**8**	F	70	S	**-**	**-**	**33**	M	38	S	**-**	**-**
**9**	F	49	S	**-**	**-**	**34**	M	69	S	**-**	**-**
**10**	M	51	S,A	**-**	**-**	**35**	M	66	S	**-**	**-**
**11**	M	70	S	**-**	**-**	**36**	F	33	S	**-**	**-**
**12**	F	33	S	**-**	**-**	**37**	F	65	S	**-**	**-**
**13**	F	19	S	**-**	**-**	**38**	F	35	S	**-**	**-**
**14**	F	53	S	**-**	**-**	**39**	F	40	S	**-**	**-**
**15**	F	22	S	**-**	**-**	**40**	F	31	S	**-**	**-**
**16**	F	22	S	**-**	**-**	**41**	F	40	S	**-**	**-**
**17**	M	30	S,A	**-**	**-**	**42**	F	41	S	**-**	**-**
**18**	F	23	S	**-**	**-**	**43**	F	24	S	**-**	**-**
**19**	F	50	S	**-**	**-**	**44**	F	24	S	**-**	**-**
**20**	F	70	S	**-**	**-**	**45**	F	35	S	**-**	**-**
**21**	M	40	S	**-**	**-**	**46**	M	27	S	**-**	**-**
**22**	M	69	S	**-**	**-**	**47**	F	54	S	**-**	**-**
**23**	M	23	S	**-**	**-**	**48**	F	31	S,A	**-**	**-**
**24**	F	70	S	**-**	**-**	**49**	F	31	S,A	**-**	**-**
**25**	M	36	S	**-**	**-**	**50**	F	29	S	**-**	**-**

“M”, male; “F”, female; “S”, sensitive; “A”, allergic.

## Data Availability

Data are contained within the article. The raw data supporting the conclusions of this article will be made available by the authors on request.
